# Medication Adherence of Children with Congenital Hypothyroidism in Iran: A National Cross-Sectional Study

**DOI:** 10.5812/ijem-150329

**Published:** 2024-08-14

**Authors:** Shahin Yarahmadi, Bahram Nikkhoo, Shiva Bararpour, Parisa Marabi, Khaled Rahmani

**Affiliations:** 1 Ministry of Health, Tehran, Iran; 2 Department of Pathology, Faculty of Medicine, Kurdistan University of Medical Sciences, Sanandaj, Iran; 3 Faculty of Medicine, Kurdistan University of Medical Sciences, Sanandaj, Iran; 4 Liver and Digestive Research Center, Research Institute for Health Development, Kurdistan University of Medical Sciences, Sanandaj, Iran

**Keywords:** Congenital Hypothyroidism, Adherence, Compliance, Medication

## Abstract

**Background:**

Congenital hypothyroidism is the most common preventable and treatable cause of intellectual disability in children. A key component of the surveillance system for congenital hypothyroidism is ensuring a regular treatment program for affected children. Despite nearly 20 years since the successful implementation of the newborn screening program for hypothyroidism in Iran, a comprehensive evaluation of patients' adherence to treatment has not been conducted.

**Objectives:**

The aim of this study was to investigate the adherence to treatment among patients with congenital hypothyroidism in Iran.

**Methods:**

In this national cross-sectional study conducted in 2024, the adherence to treatment of 400 children with congenital hypothyroidism born between 2019 and 2023 in Iran was examined using the Morisky Medication Adherence Scale. The patients were randomly selected from national registry data. Data were analyzed using chi-squared tests, Fisher's exact test, and logistic regression in Stata software version 16.

**Results:**

The mean and standard deviation of medication adherence was 6.35 ± 1.41. Overall, adherence was good (≥ 6) in 284 (71.0%) of the study participants. In the univariate analysis, the most significant factors influencing adherence were place of residence, higher maternal education, lower paternal education, and type of congenital hypothyroidism (CH). In the multivariate analysis, children with permanent CH had good adherence, and parental education was not statistically significant (P > 0.05).

**Conclusions:**

The results of this study showed that medication compliance in more than two-thirds of hypothyroid children diagnosed by national newborn screening is good. Given the importance of treatment in these patients, it is recommended that intervention plans be implemented, including educational programs and active follow-up of patients to increase compliance.

## 1. Background

Congenital hypothyroidism (CH), the most common congenital endocrine disorder, is one of the most preventable causes of mental retardation in children (1). Hypothyroidism is asymptomatic at birth in 95% of cases and rarely presents with symptoms such as swelling around the eyes, drowsiness, large tongue, and prolonged jaundice (2). Timely diagnosis of this disease is crucial because the first few years of life are critical for brain development, which depends on thyroid hormones. As mentioned, this disease rarely shows symptoms at birth. However, if diagnosed early, its irreversible and irreparable complications can be avoided. The causes of CH include disorders in the development of the thyroid gland and in the synthesis of thyroid hormones (3). Congenital hypothyroidism is one of the most significant health problems in Iran, with an average incidence of 2.6 per 1000 live births (4).

Adherence to medications is crucial for the treatment of children with CH. In addition to the early detection of CH in newborns, successful treatment outcomes are possible if parents of hypothyroid children have higher medication adherence. In fact, compliance with medication is essential to improve the neurodevelopmental outcomes of these children (5).

Early detection and timely treatment of neonates with CH are crucial, as initiating treatment before the 20th day after birth is associated with better outcomes (6). We previously assessed the physical development and intelligence quotients of hypothyroid patients diagnosed through the national newborn screening program in Iran. The results showed that the intelligence quotient and physical development of affected children at the age of 6 years were almost similar to those of healthy children of the same age (7).

According to the national guidelines for newborn screening in Iran, all children with congenital hypothyroidism should have their blood thyroid hormone levels brought within the normal range as soon as possible by prescribing Levothyroxine. The goal of the treatment is to normalize the thyroxine concentration in the baby's serum within 2 weeks and TSH within a month. The initial amount of Levothyroxine prescribed is 10 to 15 micrograms per kilogram of the baby's weight. After crushing the tablet, the powder is dissolved in water or breast milk and fed to the baby (8, 9).

The level of cooperation and treatment compliance of families of affected children is a crucial component of the success of this program. Although almost 18 years have passed since the implementation of the newborn hypothyroidism screening program in Iran (4), no study has been conducted at the provincial or national level to measure compliance with treatment for this disease. 

## 2. Objectives

Globally, studies on this topic are also very limited (10). This study aimed to assess the compliance of patients' guardians with levothyroxine therapy and explore the socioeconomic factors affecting medication adherence in patients with CH.

## 3. Methods

### 3.1. Study Design, Setting and Participants

This national cross-sectional study was conducted in 2024 on 400 parents of children with CH in eight provinces of Iran. The provinces selected for this study had a higher incidence of CH. The sample size was calculated using the sampling formula for estimating mean adherence scores. To estimate the mean adherence scores with a 5% type 1 error, a precision of 1 score on either side (more or less than the mean adherence score), a standard deviation of 1.3 (based on previous studies), and a 95% confidence interval (z = 1.96), the sample size was determined. The selected provinces include Ardabil, Alborz, Khorasan Razavi, Khuzestan, Kurdistan, Gilan, and Hamadan. Samples were randomly selected from the list of hypothyroid children in the national newborn screening registry.

### 3.2. Data Collection and Measurements

Baseline demographic and clinical data of children with CH, including sex, type of hypothyroidism (transient or permanent), and demographic data of their parents, including education, job, and place of residence, were collected using a checklist tailored to the variables and study objectives. The checklist was completed by interviewing the parents of the children. To assess the adherence rate, a translated Iranian version of the Morisky Medication Adherence Scale-8 (MMAS-8) was used. The validation and reliability of the MMAS-8 for patients with chronic diseases have been evaluated and approved (11). The Persian version of this tool has already been validated in adults with diabetes (12).

The MMAS-8 consists of seven dichotomous items and a five-point Likert scale. The questions are worded to avoid a 'yes' bias, so that each 'no' answer is worth one point, except for item 5 where the reverse is true. The eighth question has five possible answers (scores from 0 to 1, in 0.25 point increments). Finally, patients are categorized as low adherent (score < 6), moderate adherent (score 6 to < 8), and high adherent (score 8) according to the score obtained. The original authors also proposed a dichotomous cut-off of low (score < 6) vs. high/moderate adherence (score ≥ 6) to facilitate statistical analysis.

### 3.3. Ethical Considerations

The proposal for this study was reviewed and approved by the Ethics Committee of Kurdistan University of Medical Sciences, Iran (Ethics Code: IR.MUK.REC.1401.392). Written informed consent was obtained from the parents/guardians of the children before data collection. Several methods were used to protect the confidentiality, anonymity, and privacy of the participants. No personal details were included in the reports, and data were collected using assigned numbers rather than names.

### 3.4. Statistical Analysis

Data were summarized using descriptive statistics, including mean, standard deviation, frequencies, and percentages. Chi-squared tests, Fisher's exact test, and logistic regression modeling were used to examine the association between the study's independent variables and medication adherence. All analyses were performed using Stata software version 16.

## 4. Results

A total of 400 children were included in the analysis. The baseline demographic, clinical, and paraclinical characteristics of the subjects are summarized in Table 1. 

**Table 1.** Baseline Demographic Characteristics of the Study Subjects

Variables	No. (%)
**Province**	
Ardebil	25 (6.25)
Alborz	40 (10.0)
Khorasan Razavi	135 (33.75)
Khuzestan	62 (15.5)
Kurdistan	86 (21.5)
Gilan	20 (5)
Hamadan	10 (2.5)
Yazd	22(5.5)
**Gender**	
Male	232 (58.0)
Female	168 (42.0)
**Type of CH**	
Transient	341(85.25)
Permanent	59(14.75)
**Mother’s education**	
Illiterate	19 (4.75)
Elementary	140 (35.0)
Diploma	125 (31.25)
Academic	116 (29.0)
**Father’s education**	
Illiterate	9 (2.25)
Elementary	137 (34.25)
Diploma	143 (35.75)
Academic	111 (27.75)
**Mother’s occupation**	
House wife	360 (90.0)
Employee (Private sector)	6 (1.5)
Employee (Public sector)	34 (8.5)
**Father’s occupation**	
Self employed	239 (59.75)
Died	2 (0.5)
Employee (Private sector)	76 (19.0)
Employee (Public sector)	75 (18.75)
Farmer	8 (2.0)

As shown in Table 1, 58% of the children were male, and 82.5% of them had transient CH. Among the parents, 4.75% of the mothers and 2.25% of the fathers were illiterate. The mother's occupation was housewife for 90% of the participants, and the father's occupation was self-employed for 59.7% of the participants. 

**Figure 1.**
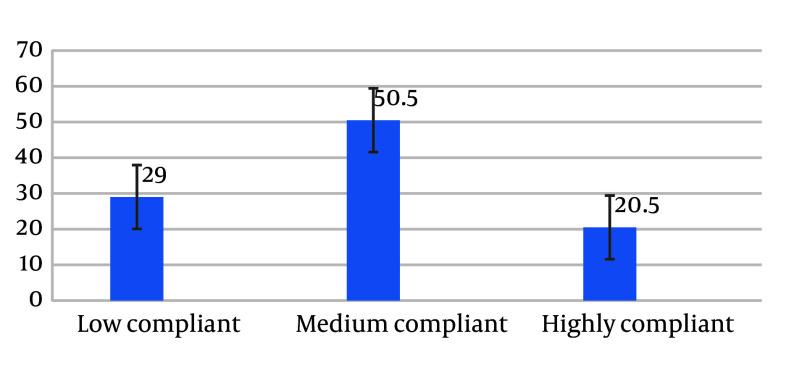
Percentage of medication compliance among the patient’s guardians

The mean and standard deviation of the adherence score by MMAS-8 was 6.35 ± 1.41 for levothyroxine medication. The results showed that medication adherence was low (< 6 score) in 116 (29.0%) children. Treatment adherence was moderate (6 - 7 score) in 202 (50.5%) children, while 82 (20.5%) children had high adherence (> 7 score) (Figure 1). Overall, adherence among 284 (71.0%) of the study subjects was good (≥ 6). The association between the study variables and treatment adherence is summarized in Table 2. 

**Table 2.** Association Between Studied Variables and Medicine Adherence (Univariate Analysis) ^a^

Variables	Adherence		P-Value
	< 6 Score, (n = 116)	≥ 6 Score, (n = 284)	
**Gender**			0.085
Male	75 (32.33)	157 (67.67)	
Female	41 (24.40)	127 (75.60)	
**Type of CH**			< 0.001
Transient	111 (32.55)	230 (67.45)	
Permanent	5 (8.47)	54 (91.53)	
**Mother’s education**			0.005
Illiterate	10 (52.63)	9 (47.37)	
Elementary	50 (35.71)	90 (64.29)	
Diploma	32 (25.60)	93 (74.40)	
Academic	24 (20.69)	92 (79.31)	
**Father’s education**			0.037
Illiterate	2 (22.22)	7 (77.78)	
Elementary	51 (37.23)	86 (62.77)	
Diploma	40 (27.97)	103 (72.03)	
Academic	23 (20.72)	88 (79.28)	
**Mother’s occupation**			0.091^b^
House wife	109 (30.28)	251 (69.72)	
Employee (Public or private sector)	7 (17.50)	33 (82.50)	
**Father’s occupation**			0.111s
Self employed	79 (31.98)	168 (68.02)	
Employee (Public or private sector)	37 (24.50)	114 (75.50)	
**Province**			< 0.001
Ardebil	5 (20.0)	20 (80.0)	
Alborz	10 (25.0)	30 (75.0)	
Khorasan Razavi	27 (20.0)	108 (80.0)	
Khuzestan	27 (43.55)	35 (56.45)	
Kurdistan	28 (32.56)	58 (67.44)	
Gilan	2 (10.0)	18 (90.0)	
Hamadan	1 (10.0)	9 (90.0)	
Yazd	16 (72.73)	6 (27.27)	

^a^ Values are expressed as No. (%).

^b^ Fisher's exact test.

As shown in Table 2, univariate analysis indicated that the variables of province of residence, parental education level, and type of congenital hypothyroidism (transient or permanent) had a significant relationship with the level of treatment compliance among the patients' families. Although not significant, adherence was higher among parents of female children (75.6%) compared to parents of boys (67.6%) (P = 0.085). The percentage of good adherence was significantly higher among parents of children with permanent CH (91.5%) compared to parents of children with transient CH (P < 0.001). Parental education was another significant factor related to medication adherence, with higher maternal education (P = 0.005) and lower paternal education (P = 0.037) being significantly associated with good adherence. Although the percentage of good compliance was higher among parents employed in the public or private sector compared to other occupations, the difference was not significant (P > 0.05). Significant differences were observed between the places of residence of hypothyroid children, with Gilan and Hamadan showing the highest adherence (90%) and Yazd the lowest (27.27%) among the studied provinces (P < 0.001). To control for potential confounders, logistic regression modeling (multivariate analysis) was conducted (Table 3). 

**Table 3.** Logistic Regression Modeling Results of Association Between Studied Variables and Medicine Adherence (Multivariate Analysis)

Variables	OR (95% CI)	P-Value
**Gender**		
Male	1	-
Female	1.26 (0.77 - 2.06)	0.361
**Province**		
Ardebil	1	-
Alborz	0.63 (0.17 - 2.28)	0.480
Khorasan Razavi	1.05 (0.34 - 3.26)	0.937
Khuzestan	0.62 (0.19 - 2.06)	0.437
Kurdistan	0.92 (0.28 - 2.96)	0.885
Gilan	3.09 (0.50 - 19.05)	0.224
Hamadan	4.88 (0.47 - 51.07)	0.186
Yazd	0.11 (0.02 - 0.45)	0.002
**CH type**		
Permanent	1	-
Transient	0.21 (0.08 - 0.58)	0.003
**Mother education**		
Illiterate	1	-
Elementary	1.58 (0.52 - 4.81)	0.422
Diploma	2.59 (0.79 - 8.48)	0.115
Academic	2.72 (0.74 - 9.94)	0.130
**Father education**		
Illiterate	1	-
Elementary	0.26 (0.03 -2.17)	0.212
Diploma	0.28 (0.03 -2.47)	0.254
Academic	0.39 (0.04 - 3.62)	0.411
**Mother occupation**		
House wife	1	-
Occupied (Public or private sector)	1.02 (0.35 - 2.96)	0.964
**Father occupation**		
Self employed	1	-
Occupied (Public or private sector)	1.31 (0.74 - 2.32)	0.351

Abbreviations: OR, odds ratio; CI, confidence interval.

As shown in Table 3, the type of CH and place of residence were two significant factors associated with adherence when modeling the data. Good adherence among children living in Yazd was significantly lower than among children living in Ardebil (OR = 0.11; P = 0.002), indicating that adherence in Yazd was 9.09 times lower. Similarly, good adherence among parents of children with transient CH was significantly lower than among parents of children with permanent CH (OR = 0.21; P = 0.003), making adherence 4.76 times lower in the transient CH group. Another important factor significantly associated with adherence in the univariate analysis was maternal education. Although not significant in the regression modeling, mothers with a university education (OR = 2.72), a diploma (OR = 2.59), and primary education (OR = 1.58) showed higher adherence compared to illiterate mothers.

## 5. Discussion

Congenital hypothyroidism is one of the most critical childhood diseases due to its potentially dangerous consequences if not treated. Normal growth and development of the fetal thyroid gland and thyroid hormone production are essential for brain development during fetal life and beyond. Besides early diagnosis and timely initiation of treatment, patient follow-up and medication adherence by the families of affected patients are crucial. The present study aimed to determine the level of medication adherence among families of patients with neonatal hypothyroidism identified in Iran. Our results showed that about two-thirds of the patient families had good medication adherence. In univariate analysis, the most significant factors influencing adherence were place of residence, higher maternal education, and type of CH, while in multivariate analysis, children with permanent CH had good adherence and parental education was not significant.

The results showed that 71.4% of children had good adherence. In a study conducted by Elshorbagy et al. in Sharkia, Egypt, two-thirds of the patient guardians were highly compliant (13), which was consistent with our findings.

The results indicated that the educational level of the mother, as the main caregiver of children with CH, is a major factor in increasing adherence to the national program for the identification and treatment of hypothyroid children. A study conducted by Brito et al. in Bahia State, Brazil, assessed the knowledge of caregivers of children with CH. Their results showed that one-third of caregivers had poor knowledge (37.3%) or were unaware (24.1%) of the importance of congenital hypothyroidism. They also showed that the low level of knowledge about the condition was related to the caregivers' level of education (10). Saber in a study in Egypt, showed that maternal education, social class, and the presence of symptoms at presentation were the main factors associated with increased treatment adherence in patients with CH (14).

Adherence to treatment and the level of parental cooperation for affected children are among the most important components of the national newborn screening for CH, given the long duration of treatment required for these patients. In a study conducted to assess the impact of medication compliance on neurodevelopmental outcomes in Sharkia Governorate, Egypt, high medication compliance was shown to be an important prognostic factor for normal mental and physical development in patients with CH (13). Another study by Bain and Toublanc concluded that the day of treatment initiation and treatment adherence were the main prognostic factors for adult height in patients with CH (15).

Our data showed that medication compliance was higher among parents of children with permanent CH compared to those with transient CH. We did not find any scientific evidence in the literature to justify this finding. It may be that the permanent nature of the disease and the increased sensitivity of the family to the child's treatment necessitate more frequent education from the treating physicians and other healthcare providers.

The present study has strengths and limitations. Based on a review of the available scientific literature, this study is the first comprehensive evaluation of adherence to treatment in children with congenital hypothyroidism at a national level in any country. Previous studies have been limited to specific regions, such as provinces in Brazil and Egypt. The use of a large sample size, direct data collection, and interviews with the children's parents are considered methodological strengths of the study. The main limitation of the study is that it did not examine the impact of patients' economic and cultural factors on medication adherence.

### 5.1. Conclusions

The results of this study showed that medication adherence is good in about two-thirds of hypothyroid children diagnosed through national newborn screening. Although not statistically significant, maternal education appears to be a main factor for higher adherence. Based on available scientific evidence, the congenital hypothyroidism screening program has been one of the successful programs of the Iranian health system over the past two decades. One of the pillars ensuring the success of this program's continued implementation is daily attention to the treatment of patients. Since nearly 30% of patients still do not adhere adequately to their therapy, and considering that in most cases the treatment period is long, it is recommended to implement intervention plans, including educational programs, along with active follow-up of patients to increase compliance.

## Data Availability

The datasets used and/or analyzed during the current study are available from the corresponding author on reasonable request.
